# Anti-Inflammatory and Antioxidant Actions of Methyl Jasmonate Are Associated with Metabolic Modifications in the Liver of Arthritic Rats

**DOI:** 10.1155/2018/2056250

**Published:** 2018-08-23

**Authors:** Anacharis B. Sá-Nakanishi, Jamil Soni-Neto, Lucas S. Moreira, Geferson A. Gonçalves, Francielli M. S. Silva, Lívia Bracht, Ciomar A. Bersani-Amado, Rosane M. Peralta, Adelar Bracht, Jurandir F. Comar

**Affiliations:** ^1^Department of Biochemistry, State University of Maringa, 87020900 Maringá-PR, Brazil; ^2^Department of Pharmacology and Therapeutics, State University of Maringa, 87020900 Maringá-PR, Brazil

## Abstract

Methyl jasmonate (MeJA) is a fatty acid-derived cyclopentanone which shares structural similarities with prostaglandins and has been under study as a promising anti-inflammatory agent. This study investigated the actions of MeJA on systemic inflammation and oxidative status in rats with adjuvant-induced arthritis, a model for rheumatoid arthritis. MeJA (75 to 300 mg·kg^−1^) was administrated orally during 18 days after arthritis induction with Freund's adjuvant. Articular and systemic inflammation was greatly increased in arthritic rats, likewise the oxidative stress in plasma and liver. The hepatic glucokinase activity and glycolysis were increased in arthritic rats. MeJA decreased most inflammatory parameters and abolished the increased protein carbonylation in plasma and liver, diminished the increased hepatic ROS content, and restored the hepatic GSH/GSSG ratio in arthritic rats. However, the MeJA treatment decreased the hepatic glucokinase activity and glycolysis and stimulated mitochondrial ROS production in healthy and arthritic rats. Oxygen uptake was increased by MeJA only in livers from treated arthritic rats. This action may bear relation to the increased activity of mitochondrial NADP^+^-dependent enzymes to provide reducing equivalents for the glutathione cycle. These beneficial effects, however, are associated with a decreased glucose flux through the glycolysis in the liver of arthritic and healthy rats.

## 1. Introduction

**Figure 1:**
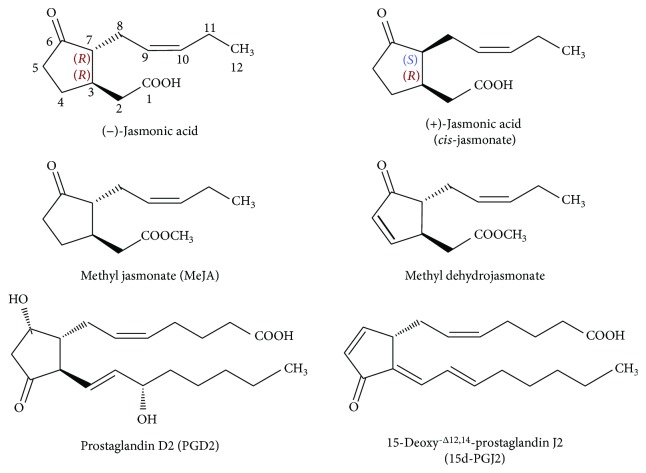
Chemical structures of jasmonates and anti-inflammatory prostaglandins. Jasmonic acid and *cis*-jasmonate differ by stereochemistry of R group at asymmetric center C-7. Methyl dehydrojasmonate is a synthetic analog of methyl jasmonate (MeJA) with an enone functional moiety. 15-Deoxy-^*Δ*12,14^-prostaglandin J2 (15-deoxy-PGJ2) is formed by two consecutive dehydration reactions of prostaglandin D2 (PGD2). The images were modified from the original ones (Wikimedia Commons, the free media repository; Files: Enantiomers Jasmonic acid.svg and Prostaglandin D2.svg) using the program CorelDRAW® Graphics Suite X7 (Corel Corporation).

Jasmonates are fatty acid-derived cyclopentanones widely distributed over the plant kingdom where they act as signaling molecules in response to abiotic and biotic stresses [1]. The jasmonate family consists mainly of jasmonic acid, *cis*-jasmonate, and methyl jasmonate (MeJA), which share structural similarities with prostaglandins, especially with those that have anti-inflammatory activities (Figure 1) [2]. For this reason, many studies have been carried out to evaluate their actions on mammal cells. Jasmonates have been reported to have cytotoxic activity against cancer cells without affecting normal cells [1, 2]. The anticancer activity of MeJA, however, showed to be superior to other jasmonates, and therefore, MeJA and its synthetic derivatives have been lately more intensely investigated as promising agents for cancer treatment [2]. MeJA induces apoptosis and inhibits proliferation in murine and human cancer cell lines, including those of breast, colon, prostate, and lymphoma [2]. In addition, MeJA increases the survival period of mice bearing the EL-4 lymphoma and of mice inoculated with multiple myeloma (MM.1S) cells [3, 4].

Mitochondria of cancer cells seem to be the main target of MeJA action, where it stimulates reactive oxygen species (ROS) production, binds to and detaches mitochondria-bound hexokinase, induces cytochrome *c* release, causes ATP depletion and finally cell death [1, 5, 6]. The association between cancer and chronic inflammation has been widely accepted and the anticancer activity of MeJA seems to be in addition related to anti-inflammatory actions [7]. Previous studies showed that MeJA inhibits the NF*κ*B-mediated production of nitric oxide (NO), prostaglandin E, TNF-*α*, IL-1, and IL-6 in lipopolysaccharide (LPS)-activated murine macrophages (RAW264.7) [8–10].

Rheumatoid arthritis is an autoimmune and chronic inflammatory disease that affects primarily the joints and occurs in 0.5–1.0% of the adult population worldwide [11]. The pathophysiology of arthritis involves intense hyperplasia of the synovial membrane and cartilage with participation of proinflammatory cytokines and overproduction of reactive species, which act as mediators of tissue injury [12]. Rheumatoid arthritis is a systemic disease and in addition to the joints other organs are affected, such as brain, heart, and lungs [12]. In addition to inflammation, oxidative stress is increased in the joints and systemically [13].

Metabolic alterations are also prominent in rheumatoid arthritis, as the muscle wasting condition known as rheumatoid cachexia [14]. Metabolic modifications are equally significant in the liver of rats with adjuvant-induced arthritis, such as increased glycolysis, reduced gluconeogenesis and altered metabolism of xenobiotics [14–16]. Oxidative stress is also altered in the plasma, liver, heart, and brain of arthritic rats [17–20]. Particularly in the liver, where inflammation and metabolic alterations are prominent, oxidative stress is quite pronounced when compared to other organs [19, 20].

Considering the above-mentioned actions of MeJA, it seems reasonable to hypothesize that it could attenuate the articular and systemic inflammation that occurs in arthritis. Anti-inflammatory actions of MeJA were mainly demonstrated in isolated cells, but *in vivo* approaches have seldom been reported. A recent study showed that increased brain levels of prostaglandin E, TNF-*α*, and IL-1 in LPS-induced neuroinflammation in mice were reduced by intraperitoneally administered MeJA, which also suppressed COX-2, iNOS and NF-*κ*B expression [21]. However, these actions refer specifically to neuroinflammation and so far no other systemic manifestation has been evaluated. Therefore, the present study aimed to investigate the action of orally administrated MeJA on the systemic inflammation and oxidative stress in rats with adjuvant-induced arthritis. The latter is an experimental immunopathology in rats which shares many features of rheumatoid arthritis and is often used as a model for evaluation of anti-inflammatory drugs [22]. Because MeJA has been reported to stimulate mitochondrial ROS production [1], this study has also evaluated the production of ROS and the respiratory activity in isolated hepatic mitochondria of healthy and arthritic rats. Furthermore, hexokinase catalyzes the first rate-limiting step of glycolysis and both glucose phosphorylation and the glycolytic flux are increased in the liver of arthritic rats [15]. These facts make it of interest to search for possible effects of MeJA on hepatic hexokinase (glucokinase) and glycolysis.

The experimental model used in the present study is considered a severe arthritis model in rats [17]. Considering that rheumatoid arthritis can range from a mild form to other more severe and disseminated forms, the present study aims to provide data about the systemic effects of MeJA in rats with polyarthritis, which in turn should also allow extrapolations to patients with rheumatoid arthritis, particularly to those that manifest the more aggressive forms.

## 2. Materials and Methods

### 2.1. Chemicals

Methyl jasmonate, o-phthalaldehyde (OPT), 2,2′-azino-bis(3-ethylbenzothiazoline-6-sulfonic acid) diammonium salt (ABTS), 6-hydroxy-2,5,7,8-tetramethylchromane-2-carboxylic acid (Trolox), 5,5-dithiobis-2-nitrobenzoic acid (DTNB), 2,4-dinitrophenylhydrazine (DNPH), oxidized dichlorofluorescein (DCF), reduced glutathione (GSH), and oxidized glutathione (GSSG) were purchased from Sigma Chemical Co. (St. Louis, MO, USA). Commercial kits for AST, ALT, alkaline phosphatase, creatinine, albumin, and total proteins were purchased from Gold Analisa Diagnóstica Ltda (Belo Horizonte, MG, Brazil). All other chemicals were of analytical grade.

### 2.2. Animals and Induction of Arthritis

Male *Holtzman* rats weighting 170–180 g were obtained from the Center of Animal Breeding of the State University of Maringá (UEM) and maintained in an Animal Care Unit of our laboratory under standard conditions of temperature (24 ± 3°C) in a regulated 12 h light/dark cycle. The animals were kept in steel cages (3 rats/cage) and were fed ad libitum with laboratory diet (Nuvilab®, Colombo, Brazil). Arthritis was induced by means of a subcutaneous injection of Freund's adjuvant in the left hind paw (0.1 mL containing 500 *μ*g of heat-inactivated *Mycobacterium tuberculosis* from H37Rv strain, suspended in Nujol®) [17]. Rats of similar weights and age served as controls. All procedures followed the guidelines of the Brazilian Council for the Control of Animal Experimentation (CONCEA) and were previously approved by the Ethics Committee for Animal Experimentation of the State University of Maringá (protocol number CEUA 6053280915).

### 2.3. Experimental Design

Forty-nine rats were randomly distributed into seven groups: controls (C), to which corn oil was administered; controls (C300) treated with MeJA at the dose of 300 mg·kg^−1^; arthritic rats (A), to which corn oil was administered; arthritic rats (A75, A150, and A300) treated with MeJA, respectively, at the doses of 75, 150, and 300 mg·kg^−1^; and arthritic rats (IBU) treated with ibuprofen at the dose of 30 mg·kg^−1^. This procedure was repeated three times (147 animals in total) to evaluate all parameters of this study. Animals were orally treated once a day with MeJA, corn oil, or ibuprofen for 5 days prior and by 18 days after the arthritis induction. Daily doses of MeJA were stipulated considering the effective dose that caused no toxicity as described elsewhere [23].

### 2.4. Evaluation of the Inflammatory Response

The volume of paws was measured daily by plethysmography (UGO BASILE SLR 21025). The severity of secondary lesions was assessed from the 10th to the 18th day as previously described [24]. Blood was collected by means of tail incision to obtain the total and differential count of circulating leukocytes. Total and differential counts of leukocytes recruited into the femorotibial joint cavity were additionally performed at the 19th day as previously described [25].

### 2.5. Blood Collection and Tissue Preparation

Fasted (12 h) rats were anesthetized with an overdose of sodium thiopental (100 mg·kg^−1^) plus lidocaine (10 mg·kg^−1^), and the peritoneal cavity was exposed. Blood was collected from the cava vein and placed into tubes with sodium heparin (100 IU mL^−1^). Next, the liver was removed and divided into two parts: one was stored in liquid nitrogen for oxidative status measurements and the other was used for mitochondria isolation. Thereafter, the hind femorotibial joints were surgically exposed, articular cavities were washed with 40 *μ*L of phosphate-buffered saline (PBS) solution containing 1 mM EDTA, and exudates were used for leukocyte count.

Blood was centrifuged (3000*g*/10 min) to separate the plasma fraction. The liver homogenate was prepared by the homogenization of a freeze-clamped tissue in a type Potter homogenizer with 10 volumes of 0.1 M potassium phosphate buffer (pH 7.4). A part of the homogenate was separated as total homogenate and the other was centrifuged (11,000*g*/15 min) for obtaining the soluble fraction of the homogenate. Fresh liver was used for mitochondria isolation by differential centrifugation as described elsewhere [26].

### 2.6. Plasma Analytical Assays

The myeloperoxidase (MPO) activity was measured spectrophotometrically with o-dianisidine (spectrophotometer Hitachi U-3000) [27]. The ferric reducing ability of plasma (FRAP) was measured by spectrophotometry (595 nm) using tripyridyltriazine (TPTZ) and ferric chloride (FeCl_3_) [28].

Protein thiol groups were measured spectrophotometrically using DTNB (5,5′-dithiobis 2-nitrobenzoic acid) as described elsewhere [17]. The molar extinction coefficient (*ε*) of 1.36 × 10^4^ M^−1^·cm^−1^ was used to calculate the thiol contents. Protein carbonyl groups were measured by spectrophotometry using 2,4-dinitrophenylhydrazine as previously described [29]. Calculations were done using the molar extinction coefficient (*ε*) of 2.20 × 10^4^ M^−1^·cm^−1^.

Albumin, total protein, creatinine, and activities of alkaline phosphatase (ALP), aspartate aminotransferase (AST), and alanine aminotransferase (ALT) were measured in the plasma to evaluate liver and kidney damage using commercial kits (Gold Analisa®).

### 2.7. Liver Oxidative Stress Parameters

Carbonylated proteins were measured spectrophotometrically in the liver homogenate supernatant with 2,4-dinitrophenylhydrazine as above described for the plasma [29].

Lipoperoxide content was measured by means of the TBARS (thiobarbituric acid reactive substance) assay [30]. The levels of TBARS were calculated from the standard curve prepared with 1,1′,3,3′-tetraethoxypropane.

The total ROS content was quantified via the 2′-7′-dichlorofluorescein diacetate (DCFH-DA) assay [31], which quantifies the oxidation of DCFH-DA to the fluorescent 2′,7′-dichlorofluorescein (DCF) in the presence of ROS. The formation of DCF was measured using a spectrofluorometer RF-5301 (Shimadzu; 504 nm for excitation and 529 nm for emission). The rate of mitochondrial ROS production (real-time ROS production) was estimated by measuring the linear fluorescence increase due to DCF formation as previously described [32]. The results were expressed as both nmol·min^−1^·(mg protein)^−1^ and the effective concentration of MeJA that stimulates ROS production half-maximally (EC_50_). EC_50_ was calculated by numerical interpolation using Stineman's interpolation formula [33].

Reduced (GSH) and oxidized glutathione (GSSG) were measured spectrofluorimetrically (excitation at 350 nm and emission at 420 nm) by means of the *o*-phthalaldehyde (OPT) assay [34]. The activities of catalase, superoxide dismutase (SOD), and myeloperoxidase (MPO) were assayed by spectrophotometry in the supernatant of the liver homogenate. The catalase activity was estimated in 240 nm using H_2_O_2_ as substrate [35]. The activity of SOD was estimated according to the pyrogallol autoxidation method [36]. MPO activity was measured as above described for the plasma [27].

### 2.8. Hepatic Glucose Phosphorylation Capacity (Glucokinase Activity)

The glucokinase activity was measured in the liquid fraction obtained by the additional high-speed centrifugation of the liver homogenate supernatant (1 h at 105,000*g*) [37]. The assay system, in a final volume of 1 mL, contained 100 mM Tris-HCl (pH 7.2), 20 mM glucose, 5 mM ATP, 10 mM MgCl_2_, 1 mM NAD^+^, 5 units of glucose 6-phosphate dehydrogenase from *Leuconostoc mesenteroides*, and 20 *μ*l of high-speed centrifugation supernatant. The increase in absorbance at 340 nm, resulting from the production of NADH, was measured during 3 min. Rates were evaluated from the slopes of the recording traces and expressed as nmol·min^−1^·(mg protein)^−1^.

### 2.9. Mitochondrial Respiratory Activity

Two protocols were used to evaluate the mitochondrial respiratory activity: (1) mitochondria isolated from animals treated with MEJA and (2) mitochondria isolated from nontreated animals incubated with exogenously added MeJA.

Mitochondrial respiration was measured by polarography using a platinum electrode [26, 38]. Mitochondria were incubated in the closed oxygraph (acrylic chamber) with a medium (2.0 mL) containing 0.25 M mannitol, 10 mM KCl, 5 mM sodium diphosphate, and 10 mM Tris-HCl (pH 7.4). Succinate and *α*-ketoglutarate, both at a concentration of 10 mM, were used as substrates. When appropriate, MeJA was added at various concentrations in the range up to 10 mM. The slopes of recorder tracings were used to calculate the rates of oxygen uptake. The respiration rates were measured under three conditions: (a) before the addition of ADP (substrate respiration or basal), (b) just after 0.125 mM ADP addition (state III respiration), and (c) after cessation of the ADP stimulation (state IV). The respiratory control (RC) was calculated as the state III/state IV ratio, and the ADP/O ratio was determined according to Chance and Williams [39].

The activities of NADH oxidase and succinate oxidase were measured polarographically using freeze-thawing disrupted mitochondria [26]. The incubation medium contained 20 mM Tris-HCl (pH 7.4), and, when appropriate, MeJA was added at various concentrations in the range up to 10 mM. The reaction was started by the addition of substrates, 1 mM NADH and 1 mM succinate, for NADH oxidase and succinate oxidase, respectively. The couple TMPD-ascorbate was in addition used as electron donating substrate to cytochrome c/complex IV of the mitochondrial respiratory chain.

### 2.10. Liver Perfusion and Glycolysis

Glycolysis was measured in the perfused livers of 12 h fasted rats. Hemoglobin-free nonrecirculating liver perfusion was performed as previously described [38, 40]. Krebs/Henseleit-bicarbonate buffer (pH 7.4) was used as perfusion liquid, saturated with oxygen by means of a membrane oxygenator. The flow was maintained constant (30 and 33 mL·min^−1^) by a peristaltic pump (Minipuls 3, Gilson, France). Glucose (20 mM) was infused during 30 minutes. Samples of the effluent perfusion fluid were collected at 2-minute intervals and analyzed for their lactate and pyruvate content [35]. Glycolysis was estimated as the sum of lactate + pyruvate production. Oxygen concentration in the venous perfusate was constantly monitored by a platinum electrode.

### 2.11. Statistical Analysis

The parameters presented in graphs and tables are means ± standard errors of the means. Statistical analysis was done by means of the GraphPad Prism Software (version 5.0). Statistical significance of the data was inferred from ANOVA one-way with Newman-Keuls post hoc testing. The 5% level of significance was adopted (*p* < 0.05). Student's *t* test was used when comparing two means (*p* < 0.05).

## 3. Results

### 3.1. Effects of MeJA on Induction and Development of Adjuvant Arthritis

Table 1 shows the inflammatory parameters due to arthritis development and the effects of methyl jasmonate treatment. The initial volume of the hind paws before adjuvant injection was 1.60 ± 0.10 mL. Inflammatory reactions in the injected paw were observed on the first day, and they were equal in all groups (not shown). At day 18, the volume of the injected paw of arthritic rats had increased by 220% relative to its initial volume. This increase was considerably less pronounced in arthritic rats treated with 300 mg·kg^−1^ MeJA (only 71%) or ibuprofen (88%). Treatments with 75 and 150 mg·kg^−1^ MeJA, however, had no effect. Arthritis increased the volume of the contralateral paw by 125%. Treatment of arthritic rats with 300 mg·kg^−1^ MeJA or ibuprofen diminished these increases to 30% and 18%, respectively. Here also the doses of 75 and 150 mg·kg^−1^ MeJA were totally ineffective. Secondary lesions appeared at day 10 and reached the highest scores at day 18. The scores at day 18 were not different in all groups.

At day 18, the number of total blood leukocytes in nontreated arthritic rats was four times higher than initially (day 0). Treatment of animals with corn oil and MeJA (75 and 150 mg·kg^−1^) did not modify the number of total leukocytes in blood, but treatment with ibuprofen and 300 mg·kg^−1^ MeJA caused a decrease by approximately 50%. The number of total leukocytes recruited into the articular cavity (joint of the injected paw) was three times higher when compared to that in the contralateral joint. Treatment of animals with ibuprofen, 150 mg·kg^−1^ MeJA, and 300 mg·kg^−1^ MeJA caused 77%, 89%, and 95% decreases, respectively. Only ibuprofen and 300 mg·kg^−1^ MeJA decreased the number of leukocytes recruited into the femorotibial right joint.

### 3.2. Biochemical Parameters in Plasma

Table 2 shows the levels of albumin, globulin, and creatinine, and the activities of MPO, AST, ALT, and ALP in the plasma. The MeJA doses of 75 and 150 mg·kg^−1^ did not affect inflammation and the corresponding data are not shown in Table 2 for simplicity. Arthritis induction increased the plasma activity of AST (+100%) and ALP (+130%). Treatment of arthritic rats with MeJA, but not with ibuprofen, maintained the AST and ALP activities at values close to the control ones. Plasma levels of creatinine were not different in rats treated or not with MeJA; however, they were 40% higher in animals treated with ibuprofen. Arthritis induction increased the activity of plasma MPO (+400%) and globulins (+30%) while it decreased the levels of albumin (−44%) and the albumin/globulin ratio (−60%). Treatment of arthritic rats with MeJA had no effects on plasma albumin, globulin, and the albumin/globulin ratio. The MPO activity, however, was approximately 40% lower when arthritic rats were treated with 300 mg·kg^−1^ MeJA or ibuprofen.

### 3.3. Oxidative Status of the Plasma

**Figure 2:**
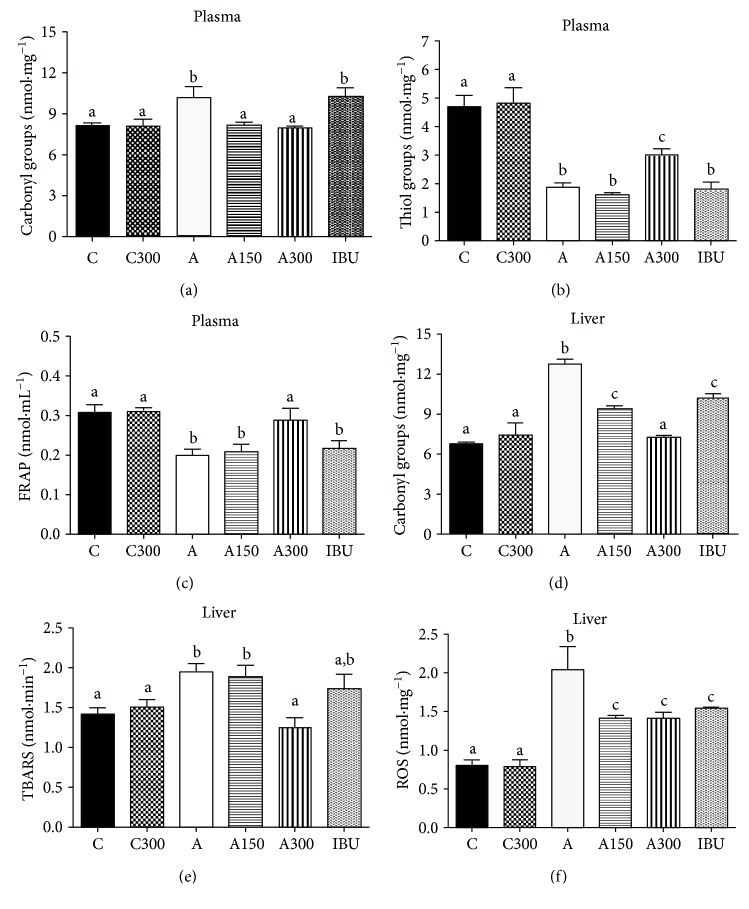
Effects of MeJA on the oxidative state of plasma and liver in arthritic rats. (a) Plasma protein carbonyl groups; (b) plasma thiol groups; (c) ferric reducing ability of plasma (FRAP); (d) hepatic protein carbonyl groups; (e) hepatic TBARS levels; (f) hepatic oxygen reactive species (ROS). C: controls treated with corn oil; C300: control treated with 300 mg·kg^−1^ MeJA; A: arthritic rats treated with corn oil; A150 and A300: arthritic rats treated with 150 and 300 mg·kg^−1^ MeJA; IBU: arthritic rats treated with ibuprofen (30 mg·kg^−1^). Data represent the mean ± SEM of 5 animals. Values with different superscript letters are statistically different (*p* < 0.05).

The levels of protein carbonyl groups, a prooxidant parameter, were 25% higher in the plasma of nontreated arthritic rats (compared to the controls; Figure 2(a)). Treatment of arthritic rats with 150 and 300 mg·kg^−1^ MeJA maintained the protein carbonyl contents at levels close to the control ones. The concentration of thiol groups in arthritic rats, an antioxidant parameter, was only 40% of that in the controls (Figure 2(b)). In arthritic animals treated with 300 mg·kg^−1^ MeJA, this decrease was attenuated, the thiol levels being 60% above those found in nontreated arthritic rats. FRAP, an antioxidant parameter, was 33% lower in nontreated arthritic rats (compared to the controls; Figure 2(c)). In arthritic animals treated with 300 mg·kg^−1^ MeJA, this decrease was diminished, the FRAP levels being 44% above those found in nontreated arthritic rats.

### 3.4. Liver Oxidative Stress

The levels of protein carbonyl groups in the liver homogenate were 80% higher in arthritic rats (Figure 2(d)). The levels of carbonyl groups were 20, 26, and 43% lower in the liver of arthritic rats treated with ibuprofen, 150 mg·kg^−1^ MeJA, and 300 mg·kg^−1^ MeJA, respectively. The levels of TBARS were 36% higher in the liver of arthritic rats (Figure 2(e)). Treatment of arthritic animals with 300 mg·kg^−1^ MeJA maintained TBARS at levels close to the control ones. The concentrations of oxygen reactive species (ROS) were 155% higher in arthritic rats (Figure 2(f)). Treatment of arthritic rats with ibuprofen, 150 mg·kg^−1^ MeJA, and 300 mg·kg^−1^ MeJA maintained the ROS contents at levels that were approximately 30% lower than that found in nontreated arthritic rats.

### 3.5. Hepatic Antioxidant and Inflammatory Status

The hepatic levels of glutathione and the activities of catalase, SOD, and MPO are shown in Table 3. No effects were found with 150 mg·kg^−1^ MeJA, and therefore, data obtained with this dose were omitted. The levels of GSH in the liver of arthritic rats were 38% lower than those in the controls. Treatment of arthritic rats with 300 mg·kg^−1^ MeJA partly prevented this decrease. The GSSG levels were similar for all groups. The GSH/GSSG ratio was 45% lower in the arthritic condition, but treatment with 300 mg·kg^−1^ MeJA prevented the decrease. The catalase activity in the liver of arthritic rats was only 20% of that in control rats, an effect that was partially prevented by the 300 mg·kg^−1^ MeJA treatment. The MPO activity was 38% higher in arthritic rats, but treatment with 300 mg·Kg^−1^ MeJA and ibuprofen totally prevented this increase.

### 3.6. Mitochondrial ROS Generation and Glucokinase Activity

**Figure 3:**
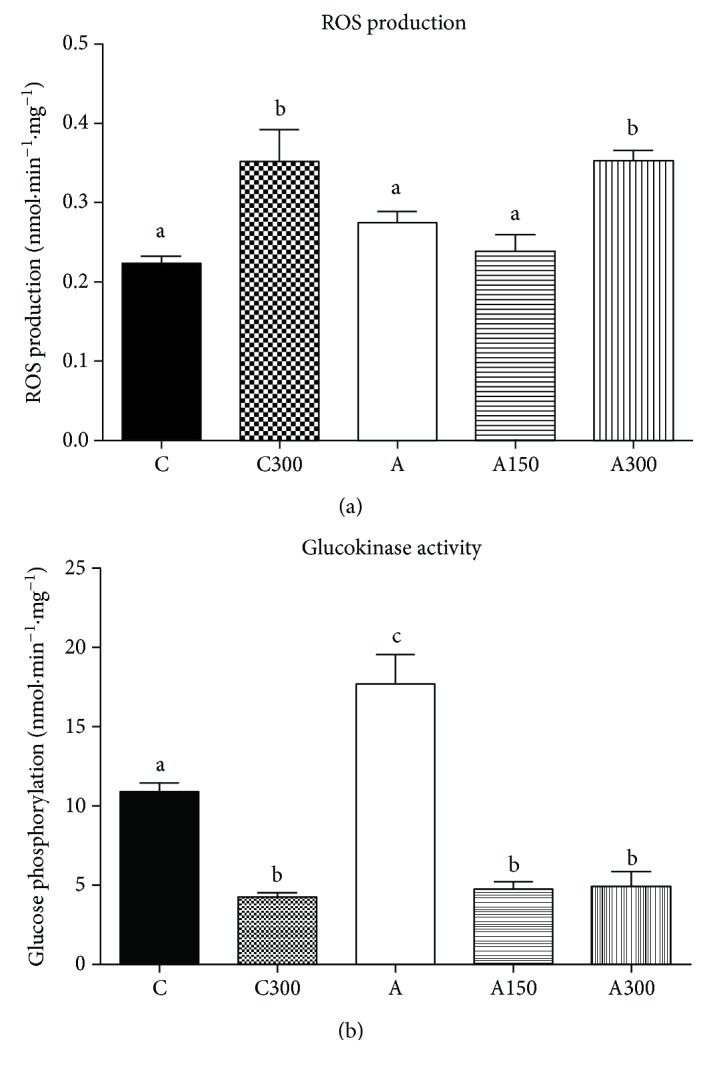
Effects of MeJA on mitochondrial ROS generation and glucokinase activity in the liver of treated rats. (a) ROS generation in isolated hepatic mitochondria and (b) glucokinase activity in liver homogenate. Isolated mitochondria and homogenate were obtained of livers from rats nontreated and treated with MeJA as described in Section 2.3. C: controls treated with corn oil; C300: control treated with 300 mg·kg^−1^ MeJA; A: arthritic rats treated with corn oil; A150 and A300: arthritic rats treated with 150 and 300 mg·kg^−1^ MeJA. Data represent the mean ± SEM of 5 animals. Values with different superscript letters in the same condition are statistically different (*p* < 0.05).

The rate of ROS production (real-time ROS production) was measured in freshly isolated hepatic mitochondria. Mitochondrial ROS production was 58 and 28% higher, respectively, in control and arthritic rats treated with 300 mg·kg^−1^ MeJA (Figure 3(a)). However, there was no difference between control and arthritic rats treated with corn oil.  Figure 3(b) shows the effects of MeJA treatment on glucokinase activity, which was 60% higher in nontreated arthritic rats (compared to the controls). Glucokinase activity was 60% lower in control rats treated with 300 mg·kg^−1^ MeJA and approximately 70% lower in arthritic rats treated with all doses of MeJA.

### 3.7. Respiratory Activity in Isolated Liver Mitochondria from MeJA-Treated Rats

**Figure 4:**
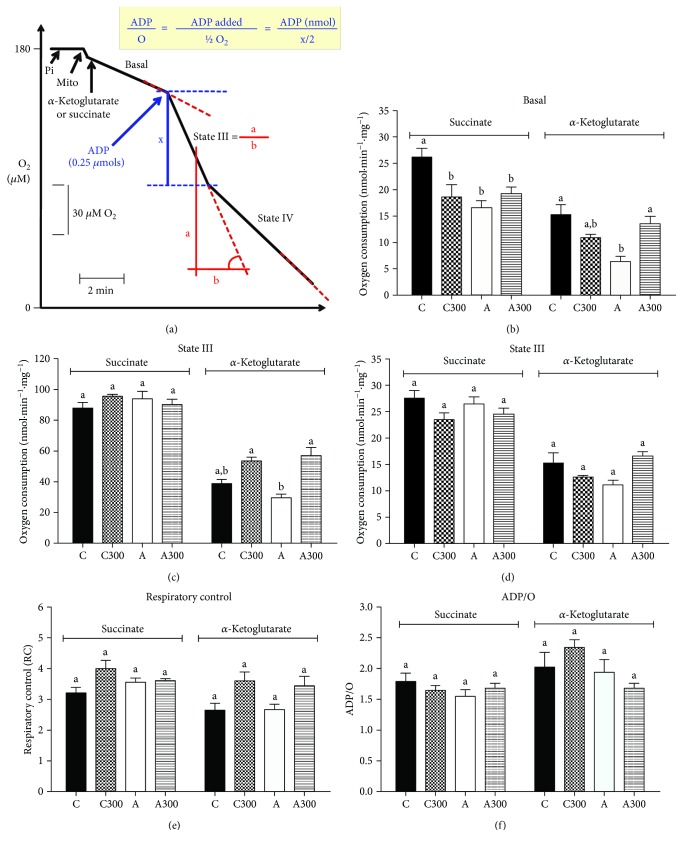
Effects of MeJA treatment on respiratory activity of intact isolated hepatic mitochondria. Hepatic mitochondria were isolated as described in Section 2.9. Succinate and alpha-ketoglutarate (10 mM) were used as respiratory substrates. Panel a shows the experimental protocol and calculation procedures. C: controls treated with corn oil; C300: control treated with 300 mg·kg^−1^ MeJA; A: arthritic rats treated with corn oil; A300: arthritic rats treated with 300 mg·kg^−1^ MeJA. Data represent the mean ± SEM of 5 animals. Values with different superscript letters are different (*p* < 0.05).

Considering that ROS production and respiratory activity are associated phenomena in mitochondria, it was evaluated if MeJA affects the respiration of isolated hepatic mitochondria.  Figure 4(a) outlines the experimental approach used to evaluate the respiratory activity of phosphorylating liver mitochondria. Basal respiration was 37% (with succinate) and 58% (with *α*-ketoglutarate) lower in mitochondria of nontreated arthritic rats (Figure 4(b)). Treatment with MeJA decreased basal respiration (−30%) in controls when succinate was the substrate but stimulated it (+110%) when *α*-ketoglutarate was the substrate. State III respiration was not modified in mitochondria from control rats treated with MeJA, but it was stimulated (+90%) in mitochondria from arthritic rats when *α*-ketoglutarate was the substrate (Figure 4(c)). State IV respiration, RC, and ADP/O ratio were not modified by MeJA treatment.

### 3.8. Glycolysis in the Perfused Liver of MeJA-Treated Rats

**Figure 5:**
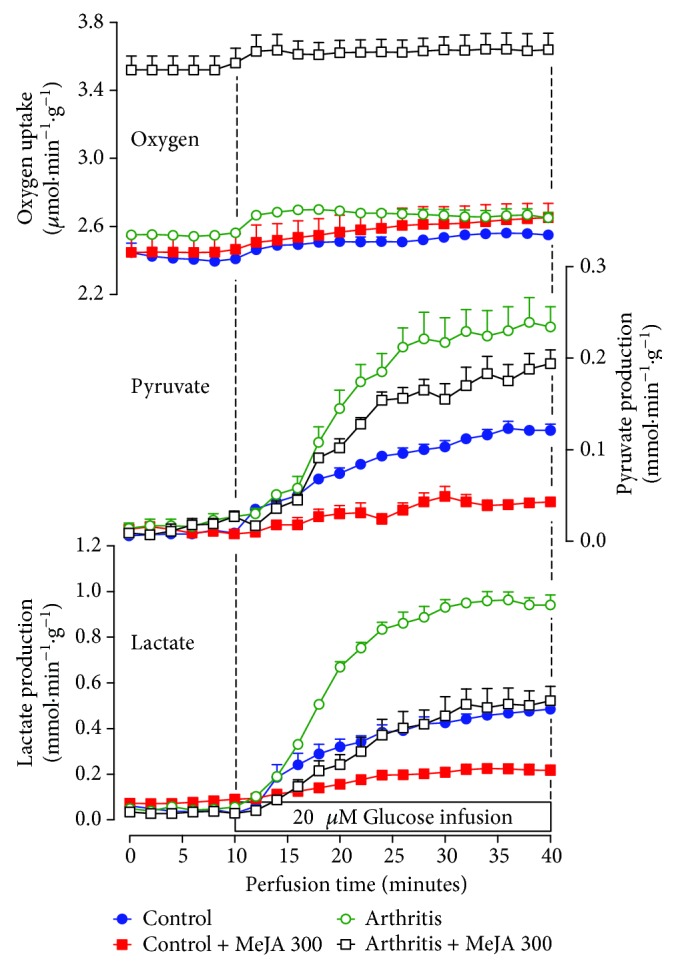
Effects of methyl jasmonate treatment on liver glycolysis and oxygen uptake. Control and arthritic rats were treated with MeJA at a dose of 300 mg·kg^−1^, as described in Section 2.3. Livers from 12 h fasted rats were perfused with Krebs/Henseleit bicarbonate buffer and 20 mM glucose as indicated by the horizontal bar. The effluent perfusate was sampled in 2 min intervals and analyzed for lactate and pyruvate. Oxygen uptake was monitored by polarography. Data are mean ± SEM obtained with 4 animals.

The diminution of the liver glucokinase activity by MeJA should lead to a decreased flux of glucose through the glycolytic pathway. To test this hypothesis, experiments with perfused livers were done to evaluate glycolysis and oxygen uptake.  Figure 5 illustrates the time courses of oxygen consumption and lactate and pyruvate production from 20 mM glucose in livers of rats treated with saline or 300 mg·kg^−1^ MeJA. The basal rates (before glucose infusion) of lactate and pyruvate production were minimal and similar in all groups, but the basal rate of oxygen consumption was more elevated in arthritic rats treated with MeJA (Figure 5). Upon the introduction of 20 mM glucose, the productions of lactate and pyruvate increased to variable extents, whereas oxygen uptake suffered relatively small and similar increments. In order to compare the groups, numerical values for each parameter at a 38-minute perfusion time in Figure 5 (steady-state) are displayed in Table 4. Treatment of control or arthritic rats with corn oil produced no differences. Lactate and pyruvate production rates were approximately 100% higher in arthritic rats. Lactate production was 50% lower in the liver of control and arthritic rats treated with MeJA. Pyruvate production was 65 and 20% lower, respectively, in control and arthritic rats treated with MeJA. The glycolytic flux (lactate + pyruvate/2) was modified in the same proportion as lactate production.

### 3.9. Mitochondrial ROS Generation and Glucokinase Activity *In Vitro*

**Figure 6:**
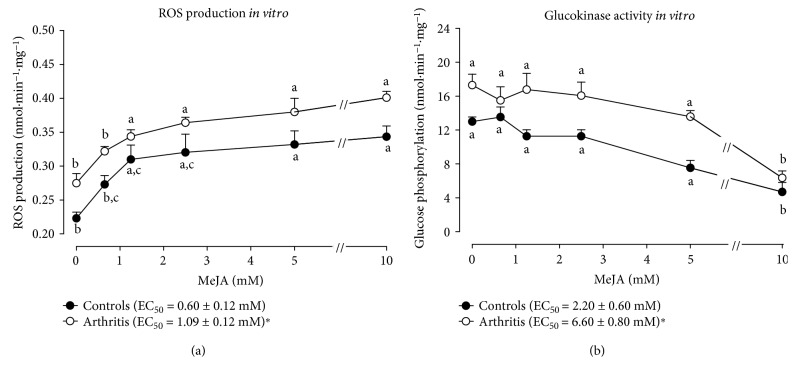
Effects of MeJA on mitochondrial ROS generation and hepatic glucokinase activity in vitro. (a) Concentration dependence of stimulation of mitochondrial ROS production by exogenously added MeJA; (b) concentration dependence of inhibition of glucokinase activity. ROS production was measured in isolated hepatic mitochondria and glucokinase activity in the supernatant of liver homogenate. EC_50_ was calculated by numerical interpolation. Data represent the mean ± SEM of 5 animals. Values with different superscript letters in the same condition are different (*p* < 0.05). EC_50_: ^∗^*p* < 0.05 for difference between control and arthritis.

Figure 6(a) shows the concentration dependence of exogenously added MeJA on mitochondrial ROS generation *in real time*. These experiments were done in order to detect possible reversible short-term effects of MeJA that are no longer present once the mitochondria or the glucokinase of treated rats is isolated from the liver cells of the intact organism. Figure 6(a) reveals that in the absence of MeJA, ROS production was 23% higher in arthritic rats (*p* = 0.014). MeJA stimulated ROS production in both mitochondria from control and arthritic rats. The final increment was approximately the same, 0.116 and 0.124 nmol·mL^−1^·mg^−1^ for the control and arthritic conditions, respectively. However, at low concentrations mitochondria from control rats were more sensitive, as can be judged from the lower concentration producing 50% stimulation (see Figure 6(a)).

Figure 6(b) shows the effects of exogenously added MeJA on the glucokinase activity in the control and arthritic conditions. The glucokinase activity in the absence of MeJA was 33% higher in the arthritic condition (*p* = 0.0225). Addition of MeJA diminished glucokinase activity in both conditions, but only at the high concentration of 10 mM.

### 3.10. Mitochondrial Respiratory Activity *In Vitro*

**Figure 7:**
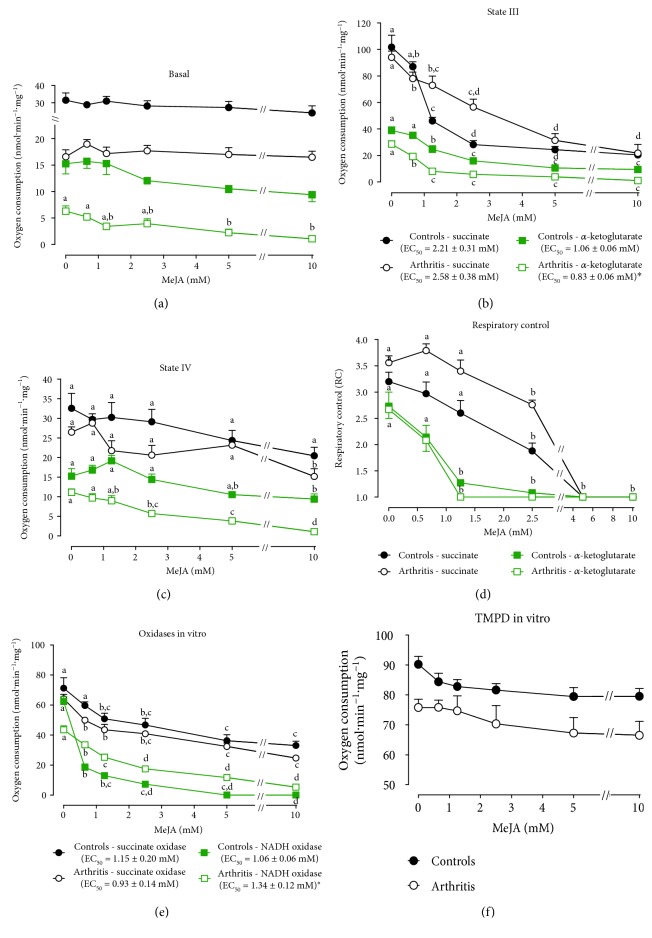
Effects of exogenous MeJA on respiratory activity of isolated hepatic mitochondria. Concentration dependence of the inhibition of basal respiration (a), state III (b), state IV (c), RC (d), mitochondrial membrane-bound enzyme activities (e), and mitochondrial respiration with TMPD-ascorbate (f) by exogenously added MeJA. Data represent the mean ± SEM of 5 animals. Values with different superscript letters in the same condition are different. EC_50_: ^∗^*p* < 0.05 for difference between control and arthritis.

The MeJA treatment of rats caused some minor modifications in the respiratory activity of mitochondria that persisted after isolation. Reversible and short-term effects can be detected in experiments where MeJA is added to mitochondria obtained from animals that were not treated with the compound. The experimental approach used to evaluate the respiratory activity of mitochondria was that one shown in Figure 4(a). MeJA was added in the range up to 10 mM. Basal respiration in the absence of MeJA was approximately 50% lower in mitochondria of arthritic rats (*p* < 0.05). MeJA decreased basal respiration only in the arthritic condition and with the substrate *α*-ketoglutarate (Figure 7(a)). State III respiration was diminished in a concentration-dependent manner irrespective of the substrate (succinate or *α*-ketoglutarate) and the conditions (healthy or arthritic; Figure 7(b)). EC_50_ of MeJA for the inhibition of state III respiration was 22% lower for arthritic rats when compared to the controls. The state IV respiration was inhibited to a lesser degree when compared to state III (Figure 7(c)). The mitochondrial respiratory control (RC), on the other hand, was diminished and even abolished with increasing MeJA concentrations for both conditions and substrates (Figure 7(d)).

Considering that the mitochondrial respiratory activity, especially state III respiration, was inhibited when incubated with MeJA, the NADH and succinate oxidase activities were further measured in disrupted mitochondria. The results are shown in Figure 7(e). Both NADH oxidase and succinate oxidase activities were progressively diminished with increasing MeJA concentrations in disrupted mitochondria from both control and arthritic rats. The EC_50_ values for succinate oxidase inhibition were similar for the control and arthritic condition, but the EC_50_ value for NADH oxidase inhibition for the control condition was only one third of that for the arthritic condition (Figure 7(e)). However, it is important to highlight that the NADH oxidase activity in the absence of MeJA was already 30% lower in arthritic rats (*p* = 0.0036; compared to the controls).  Figure 7(f) shows the effects of MeJA on oxygen consumption of disrupted mitochondria using the combination TMPD-ascorbate as substrate. Under this condition, oxygen consumption was not modified by MeJA in both the control and arthritic conditions.

## 4. Discussion

### 4.1. The Anti-Inflammatory Action

Rats with adjuvant-induced arthritis present generalized inflammatory manifestations, particularly on the 18th day after arthritis induction, when an intense inflammatory response to the adjuvant is observed in all paws (polyarthritis) in addition to leukocytosis and high levels of systemic proinflammatory cytokines [17, 20]. In this study, MeJA was effective as anti-inflammatory, notably at the dose of 300 mg·kg^−1^. The anti-inflammatory activity of MeJA was already shown previously in LPS-activated murine macrophages (RAW264.7) [8–10] and *in vivo* in the brain of mice with LPS-induced neuroinflammation [21]. Our results reveal that MeJA is additionally effective on articular and systemic inflammation, especially in the liver of rats with adjuvant-induced arthritis. Several results of this study have been included in a Master Dissertation presented at the University of Maringá [41].

The anti-inflammatory mechanism of MeJA has not yet been completely clarified, but it is known that it suppresses NF-*κ*B-mediated expression of proinflammatory enzymes and cytokines. MeJA inhibited the production of prostaglandin E, TNF-*α*, IL-1, and IL-6 as well as the expression of iNOS, COX-2, and NF-*κ*B in the brain of mice with LPS-induced neuroinflammation and LPS-stimulated RAW267.4 cells [8, 21]. In addition, MeJA was reported to attenuate activation of NF-*κ*B by suppressing degradation of its inhibitor I kappa B-alpha (I*κ*B-*α*) [21]. Evidence from studies with methyl dehydrojasmonate, a structural analog of MeJA, suggests that MeJA may in addition downregulate miR-155, which is elevated in synovial fibroblasts from rheumatoid arthritis patients and LPS-stimulated RAW267.4 cells [9, 42].

Although the effective dose of MeJA (300 mg·kg^−1^) may be considered elevated for clinical studies, it is similar to those previously used to evaluated the antitumoral properties (50–1000 mg·kg^−1^) [2]. Moreover, a notable absence of MeJA toxicity was reported for doses of this range, including an intravenous dose of 236 mg·kg^−1^ in mice [2, 3, 23]. In addition, plasma markers of hepatic and renal damage were not modified by the treatment.

### 4.2. The Antioxidant Action

The oxidative stress is systemically altered in rats with adjuvant-induced arthritis [17–20] as confirmed in the present study for plasma and liver. In the plasma, higher levels of carbonyl groups indicate that oxidative stress is occurring in a place where antioxidant enzymes and glutathione contribute poorly and the antioxidant activity depends mainly on the thiol groups of albumin [17]. MeJA decreased oxidative stress in the plasma of arthritic rats, but it did not increase the albumin levels. On the other hand, it increased the thiol groups on albumin and, consequently, the plasma antioxidant capacity.

**Figure 8:**
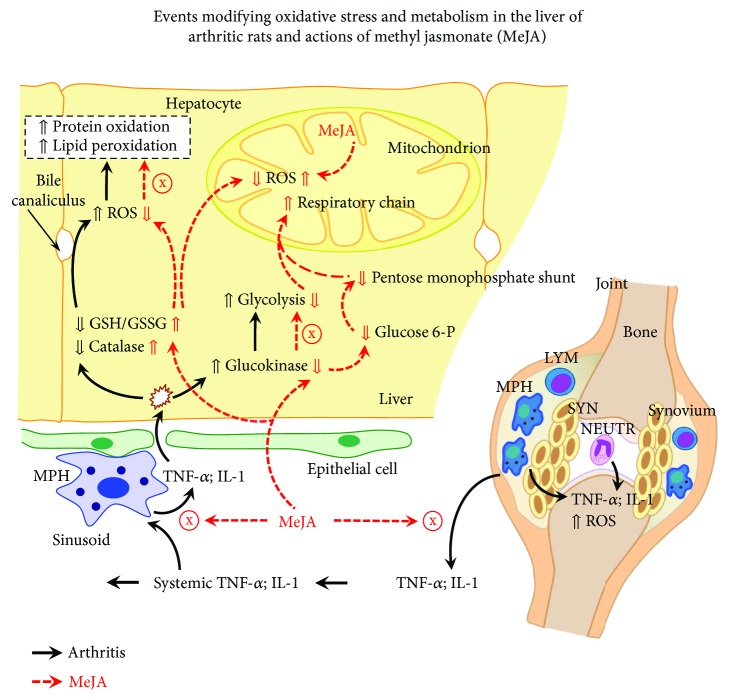
Schematic representation of the events modifying oxidative stress and metabolism in the liver of arthritic rats and actions of methyl jasmonate (MeJA). The scheme is discussed in the text and is based on the results of the current work and on previously published data. The symbol ⇑ means upregulation and ⇓ downregulation. Black arrows indicate events in the absence of MeJA, and red arrows indicate the effects of MeJA. TNF-*α*: tumor necrosis factor alpha; IL-1: interleukin 1; GSH: reduced glutathione; GSSG: oxidized glutathione; MPH: macrophages; LYM: lymphocytes; NEUTR: neutrophils; SYN: synoviocytes; ROS: reactive oxygen species.

Oxidative stress in the liver has been reported to be more pronounced when compared to other tissues in arthritic rats [19, 20]. In this study and in previous reports higher levels of ROS, lipoperoxides and carbonyl groups in the arthritic liver were accompanied by a marked deficiency in catalase and a very low GSH/GSSG ratio [20, 38]. Altered oxidative stress has been attributed to both an impaired ROS scavenging system and an increased production of ROS. Both phenomena are probably mediated by proinflammatory cytokines. In fact, TNF-*α* and IL-1 were reported to stimulate mitochondrial ROS production and to diminish catalase activity in the liver [43, 44]. These cytokines, in addition, stimulate oxidative metabolism in the liver of arthritic rats, a phenomenon that generates a more oxidizing environment and more intense production of ROS [15, 16, 20]. The very low GSH/GSSG ratio reflects the more oxidized state of the arthritic liver.  Figure 8 summarizes the key events determining oxidative stress in the arthritic liver (black arrows) and also suggests how MeJA could be acting to reverse them (red arrows). For the latter, there are two possible mechanisms: by decreasing the inflammatory process or by stimulating the endogenous antioxidant system. Both phenomena occurred in arthritic rats, as indicated, for example, by the lower MPO activity in the plasma and liver associated with the increased catalase activity and GSH/GSSG ratio in the liver, which can at least in part be attributed to an inhibition of proinflammatory cytokines. On the other hand, the cellular redox homeostasis is regulated mainly by nuclear factor erythroid 2-related 2 (Nfr2), a redox-sensible transcription factor which upregulates antioxidant defense genes, including catalase and enzymes required for GSH synthesis and regeneration [45]. Nfr2 in turn is regulated mainly by microRNAs (miRNAs), which mediate posttranscriptional gene modulation [46]. miR-155 is reported to downregulate Nfr2 while miR-101 upregulates it [46, 47]. Methyl dehydrojasmonate suppresses miR-155 induction in LPS-stimulated RAW264.7 cells and MeJA enhances the induction of miR-101 in human colorectal and bladder cancer cells [48, 49]. Therefore, MeJA may be improving the antioxidant status of arthritic rats by downregulating miR-155 and upregulating miR-101, which results in upregulation of the Nfr2 activity.

### 4.3. The Mitochondrial ROS Production and Metabolism

MeJA's cytotoxic activity against cancer cells is reported to occur as consequence of one or more of the following events: (I) increased ROS production in mitochondria of cancer cells, which are more sensitive than normal cells to higher ROS concentrations [2], and (II) modification of the mitochondrial respiration of cancer cells which provokes ATP depletion [1]; and (III) impaired glucose metabolism through glycolysis, due to hexokinase inhibition [50]. The results of this study show that hepatic cells of both healthy and arthritic rats respond to MeJA, at least partly, in the same way as cancer cells. In hepatic mitochondria of healthy and arthritic rats, the MeJA treatment stimulated ROS production over a concentration range that was similar to that in cancer cells [51]. This increased capacity of generating ROS in isolated mitochondria, however, did not result in increased oxidative stress in the healthy liver. In the liver of arthritic rats, a diminution of oxidative stress was even found upon MeJA treatment. This combination, the induction of a higher capacity of producing ROS in mitochondria and a diminished oxidative stress suggests strongly that the effects of MeJA as a stimulator of the ROS scavenging mechanisms predominate over its actions as a stimulator of ROS production in the liver of both arthritic and healthy rats. This is not what happens in cancer cells in which the net balance of the action of MeJA seems to favor ROS production [2].

Increased mitochondrial ROS production is normally associated with modifications in the mitochondrial respiration. The latter was slightly increased in mitochondria isolated after MeJA treatment of arthritic rats when *α*-ketoglutarate was the substrate in the absence and presence of exogenously added ADP (Figures 4(b) and 4(c)), but not when succinate was the substrate. As the ADP/O ratio was not modified, the small increase in state III respiration may also represent a small increase in the rate of ADP phosphorylation under these conditions. It is unlikely, however, that this small stimulation of the *α*-ketoglutarate respiration and of possibly other NADH-dependent substrates within the cell can represent significant increases in ROS generation. Moreover, it should be recalled that when MeJA was added to liver mitochondria of healthy or arthritic rats not treated with this compound, a concentration-dependent inhibition of state III respiration was found (Figure 7(b)). It is difficult to infer if this short-term inhibition is significant *in vivo* as relatively high concentrations at the mitochondrial site are required (around or above 1 mM). If this effect also occurs *in vivo* with doses up to 300 mg·kg^−1^ MeJA, however, a situation may arise in which both stimulatory and inhibitory effects match each other so that the net effect is negligible. Consequently, stimulation of mitochondrial ROS generation by MeJA is likely to occur by another mechanism that is not dependent on the respiratory activity. In cancer cells, when attached to the mitochondrial outer membrane, hexokinase decreases the mitochondrial membrane potential (Δ*ψ*_m_) and suppresses ROS generation [52]. MeJA is reported to bind hexokinase and detach it from mitochondria, a condition that stimulates ROS production in cancer cells [6]. This is a mechanism that could explain, partly at least, the increased ROS production found in mitochondria from healthy and arthritic rats treated with MeJA.

Detachment of hexokinase from the mitochondrial outer membrane is associated with a reduced capacity of glucose phosphorylation and consequently reduced flux of glucosyl units through the glycolytic pathway. The MeJA-induced glucokinase inhibition is reported to be more harmful to cancer cells than to healthy cells because the former rely more on the glycolytic pathway [6, 50]. However, the inhibition of hexokinase and even glycolysis by MeJA was measured in isolated cells [6, 50]. In the present study, hepatic hexokinase was additionally inhibited in healthy and arthritic rats treated with MeJA. Moreover, the glucose flux through the glycolytic pathway was substantially decreased in perfused livers of healthy and arthritic rats, a phenomenon that may affect energy metabolism of these cells. Additionally, it may also lead to hyperglycemia, a possibility that remains to be verified in future experiments.

The accelerated oxygen consumption in perfused livers of MeJA-treated arthritic rats deserves additional comments. When glucose oxidation is decreased, an increase in oxygen consumption would not be a surprise because a compensatory increase in the oxidation of fatty acids and amino acids could be occurring. However, this phenomenon did not occur in healthy rats treated with MeJA, an observation that speaks against the expectation of a compensatory effect on respiration due to an inhibited glycolysis. On the other hand, the difference between livers from healthy and arthritic rats could be related to different needs in terms of the corresponding antioxidant defenses. Phosphorylation of glucose is also essential for its oxidation in the pentose monophosphate shunt and to provide reducing equivalents as NADPH to the glutathione cycle. If the concentration of glucose 6-phosphate is decreased, the NADPH/NADP^+^ and, consequently, the GSH/GSSG ratios will also decrease. However, oxidative stress was improved and the GSH/GSSG ratio was increased in MeJA-treated arthritic rats. This suggests the activation of a further source of reducing equivalents. NADPH generation can occur additionally via mitochondrial isocitrate dehydrogenase 1 (IDH1) and cytosolic NADP^+^-dependent malic enzyme, which are both upregulated by Nfr2 [45]. Oxidation of fatty acids in the Krebs cycle can propel NADPH generation via isocitrate dehydrogenase and by consequence to increase hepatic oxygen uptake. In this sense, Nfr2 has been reported also to enhance mitochondrial fatty acid oxidation [45]. Thus, the accelerated oxygen uptake only in perfused livers of MeJA-treated arthritic rats could be representing an increased fatty acid oxidation for generating NADPH and not for compensating the inhibition of the glycolytic pathway.

## 5. Conclusions

In conclusion, MeJA decreased the articular and systemic inflammation in rats with severe adjuvant-induced arthritis and decreased the pronounced oxidative stress in the plasma and liver of arthritic rats. This latter effect occurs in consequence of reduced inflammation associated with an improvement of the antioxidant defenses. MeJA induced mitochondrial ROS production and inhibited the glucokinase activity in livers from healthy and arthritic rats, but it did not increase the hepatic oxidative stress. It is apparent that the effects of MeJA as a stimulator of the ROS scavenging mechanisms predominate over its actions as a stimulator of ROS production. However, the MeJA-induced glucokinase inhibition decreased substantially the flux through the glycolytic pathway of healthy and arthritic livers. In addition, the effective doses of MeJA in the present study were neither hepatotoxic nor nephrotoxic, a phenomenon that makes this compound a potentially important starting point for the development of anti-inflammatory and antirheumatic drugs. Finally, future approaches should consider the role of Nfr2 in the MeJA actions and possible modifications in systemic metabolism due to inhibition of hexokinase and hepatic glycolysis.

## Figures and Tables

**Table 1:** Inflammatory parameters and number of blood and articular leukocytes. The number of leukocytes in the peripheral blood was measured before adjuvant induction (initial, at day 0) for the arthritic group (A) and at day 18 for all groups. The number of articular leukocytes was measured in the femorotibial hind joints at day 18. The initial paw volume was 1.6 ± 0.1 ml; *Δ*vol of paws (paw edema) = volume at day 18 − initial volume. The score of secondary lesions (arthritic score) is defined in Section 2.4. A: nontreated arthritic rats; A corn oil: arthritic rats treated with corn oil; A75, A150, and A300: arthritic rats treated with 75, 150, and 300 mg·kg^−1^ MeJA; IBU: arthritic rats treated with 30 mg·kg^−1^ ibuprofen.

Parameters	Initial	A	A corn oil	A75	A150	A300	IBU
*Δ*vol − injected paw (mL)	—	4.0 ± 0.3^a^	3.5 ± 0.2^a^	4.4 ± 0.3^a^	4.0 ± 0.2^a^	1.3 ± 0.2^b^	1.6 ± 0.2^b^
*Δ*vol − noninjected (mL)	—	2.1 ± 0.3^a^	2.0 ± 0.2^a^	2.9 ± 0.2^a^	2.6 ± 0.1^a^	0.5 ± 0.1^b^	0.3 ± 0.1^b^
Arthritic score	—	5.0 ± 0.0^a^	5.0 ± 0.0^a^	5.0 ± 0.0^a^	4.4 ± 0.2^a^	4.4 ± 0.4^a^	3.5 ± 0.3^a^
	(at day 0)	Blood leukocytes (at day 18)					
Total leukocytes (×10^3^) (mm^3^)^−1^	13.1 ± 0.5^a^	66.7 ± 9.9^b^	47.2 ± 1.3^b^	52.8 ± 6.4^b^	52.8 ± 6.4^b^	32.6 ± 2.4^c^	34.6 ± 2.2^c^
PMN cells (%)	15 ± 2^a^	70 ± 3^b^	64 ± 4^b^	66 ± 3^b^	66 ± 3^b^	48 ± 6^c^	62 ± 5^b^
		Articular leukocytes (hind left joint)					
Total leukocytes (×10^4^) (mm^3^)^−1^	—	16.8 ± 3.2^a^	13.7 ± 1.7^a^	16.1 ± 1.8^a^	2.0 ± 0.7^b^	0.9 ± 0.3^c^	3.9 ± 1.1^b^
PMN cells (%)	—	53 ± 3^a^	69 ± 6^a^	62 ± 2^a^	58 ± 23^b^	61 ± 3^b^	65 ± 3^b^
		Articular leukocytes (hind right joint)					
Total leukocytes (×10^4^) (mm^3^)^−1^	—	6.9 ± 0.7^a^	6.1 ± 1.5^a^	6.5 ± 0.7^a^	7.0 ± 1.5^a^	2.3 ± 0.2^b^	1.1 ± 0.4^b^
PMN cells (%)	—	53 ± 1^a^	61 ± 8^a^	69 ± 2^a^	60 ± 1^a^	74 ± 3^a^	69 ± 4^a^

Data are mean ± SEM of 5 animals. Values with different superscript letters in the same line are different.

**Table 2:** Plasma parameters of inflammation and liver/renal damage. C, controls treated with corn oil; C300, controls treated with MeJA at the dose of 300 mg·kg^−1^; A: arthritic rats treated with corn oil; A300: arthritic rats treated with MeJA at the dose of 300 mg·kg^−1^; IBU: arthritic rats treated with ibuprofen at the dose of 30 mg·kg^−1^.

Parameter	Groups				
	C	C300	A	A300	IBU
AST (U·L^−1^)	37.6 ± 1.6^a^	36.6 ± 3.9^a^	77.4 ± 2.8^b^	52.4 ± 1.6^a^	60.6 ± 3.6^c^
ALT (U·L^−1^)	18.9 ± 0.8^a^	18.6 ± 0.6^a^	16.5 ± 1.1^a^	19.0 ± 0.6^a^	26.1 ± 0.6^a^
ALP (U·L^−1^)	60.7 ± 7.7^a^	59.0 ± 5.4^a^	139 ± 11^b^	81.1 ± 8.1^a^	151 ± 5.9^b^
Total protein (g·dL^−1^)	5.9 ± 0.3^a^	5.8 ± 0.3^a^	5.9 ± 0.1^a^	5.6 ± 0.1^a^	5.8 ± 0.2^a^
Albumin (g·dL^−1^)	2.3 ± 0.2^a^	2.3 ± 0.1^a^	1.3 ± 0.1^b^	1.6 ± 0.1^b^	2.1 ± 0.1^a^
Globulin (g·dL^−1^)	3.5 ± 0.5^a^	3.4 ± 0.1^a^	4.6 ± 0.1^b^	3.9 ± 0.2^a^	3.7 ± 0.2^a^
Albumin/globulin (A/G ratio)	0.7 ± 0.1^a^	0.7 ± 0.1^a^	0.3 ± 0.01^b^	0.4 ± 0.04^b^	0.6 ± 0.02^a^
Creatinine (mg·dL^−1^)	0.4 ± 0.1^a^	0.4 ± 0.1^a^	0.6 ± 0.01^a^	0.6 ± 0.02^a^	0.8 ± 0.1^b^
MPO activity (nmol·min^−1^·mg^−1^)	2.3 ± 0.3^a^	3.1 ± 0.2^a^	14.7 ± 1.4^b^	8.4 ± 1.1^c^	8.3 ± 0.8^c^

The data are the mean ± standard error of the mean of 5 animals. Values with different superscript letters in the same line are different.

**Table 3:** Effects of MeJA on antioxidant and inflammation parameters in the liver. C: controls treated with corn oil; C300: control treated with MeJA at the dose of 300 mg·kg^−1^; A: arthritic rats treated with corn oil; A300: arthritic rats treated with MeJA at the dose of 300 mg·kg^−1^; IBU: arthritic rats treated with ibuprofen at the dose of 30 mg·kg^−1^, GSH: reduced glutathione; GSSG: oxidized glutathione; SOD: superoxide dismutase; MPO: myeloperoxidase.

Parameter	Groups				
	C	C300	A	A300	IBU
GSH (nmol·mg^−1^)	11.4 ± 0.6^a^	12.0 ± 1.5^a^	7.2 ± 0.3^b^	12.3 ± 0.8^a^	8.7 ± 0.7^b^
GSSG (nmol·mg^−1^)	1.5 ± 0.2^a^	1.3 ± 0.1^a^	1.7 ± 0.2^a^	1.4 ± 0.3^a^	1.6 ± 0.2^a^
GSH + 2GSSG (nmol GSH units·mg^−1^)	14.3 ± 0.8^a^	14.6 ± 1.5^a^	10.5 ± 0.3^b^	14.1 ± 0.5^a^	12.0 ± 0.4^a,b^
GSH/GSSG ratio	8.7 ± 0.2^a^	10.8 ± 1.2^a^	4.8 ± 0.4^b^	8.2 ± 1.1^a^	6.5 ± 1.0^a,b^
Catalase activity (mmol·min^−1^·mg^−1^)	1.1 ± 0.09^a^	1.0 ± 0.02^a^	0.2 ± 0.02^b^	0.4 ± 0.04^c^	0.3 ± 0.03^b^
SOD activity (U·mg^−1^)	1.9 ± 0.1^a^	2.1 ± 0.2^a^	1.7 ± 0.2^a^	2.2 ± 0.2^a^	2.4 ± 0.3^a^
MPO activity (nmol·min^−1^·mg^−1^)	16.7 ± 0.1^a^	16.0 ± 0.1^a^	23.1 ± 0.8^b^	16.9 ± 0.1^a^	18.4 ± 1.6^a^

The data are the mean ± standard error of the mean of 5 animals. Values with different superscript letters in the same line are different.

**Table 4:** Effects of MeJA treatment on glycolysis and oxygen uptake in livers from control and arthritic rats. Livers from 12 h fasted rats were perfused with Krebs/Henseleit bicarbonate as described in Section 2.10. Glucose (20 mM) was infused as glycolytic substrate. The rates of lactate and pyruvate production were computed at a 38-minute perfusion time in Figure 5 (steady-state). Glycolysis was calculated as (lactate + pyruvate)/2 and expressed as *μ*mol glucosyl units·min^−1^·g^−1^. *Δ*Oxygen consumption is the increment in the oxygen consumption due to glucose infusion at a 38-minute perfusion time. C: controls treated with saline; C corn oil: controls treated with corn oil; C300: controls treated with 300 mg·kg^−1^ MeJA; A: arthritic rats treated with saline; A corn oil: arthritic rats treated with corn oil; A300: arthritic rats treated with 300 mg·kg^−1^ MeJA.

Groups	Parameter (*μ*mol·min^−1^·g^−1^)			
	Lactate production	Pyruvate production	Glycolysis	*Δ*Oxygen consumption
C	0.48 ± 0.02^a^	0.12 ± 0.01^a^	0.30 ± 0.01^a^	0.16 ± 0.03^a^
C corn oil	0.36 ± 0.03^a^	0.13 ± 0.02^a^	0.26 ± 0.02^a^	0.10 ± 0.05^a^
C300	0.22 ± 0.03^b^	0.04 ± 0.01^b^	0.13 ± 0.01^b^	0.13 ± 0.02^a^
A	0.94 ± 0.03^c^	0.24 ± 0.03^c^	0.59 ± 0.03^c^	0.12 ± 0.03^a^
A corn oil	0.84 ± 0.07^c^	0.18 ± 0.02^a,c^	0.50 ± 0.03^c^	0.29 ± 0.07^a^
A300	0.50 ± 0.06^a^	0.19 ± 0.02^a,c^	0.35 ± 0.04^a^	0.27 ± 0.09^a^

The data are the mean ± standard error of the mean of 4 animals. Values with different superscript letters in the same line are different.

## Data Availability

The data used to support the findings of this study are available from the corresponding author upon request.

## Abbreviations

MeJA: Methyl jasmonate
ROS: Reactive oxygen species
GSH: Reduced glutathione
GSSG: Oxidized glutathione
TNF-*α*: Tumor necrosis factor alpha
IL-1: Interleukin 1
COX-2: Cyclooxygenase 2
iNOS: Inducible nitric oxide synthase
NF*κ*B: Nuclear factor kappa B
Nfr2: Nuclear factor erythroid 2-related 2
miR-101 and 155: microRNAs 101 and 155
TBARS: Thiobarbituric acid reactive substances
GK: Glucokinase
G6Pase: Glucose 6-phosphatase
SOD: Superoxide dismutase
MPO: Myeloperoxidase
IDH1: Isocitrate dehydrogenase 1
FRAP: Ferric reducing activity of plasma
ALT: Alanine aminotransferase
AST: Aspartate aminotransferase
MPH: Macrophages
LYM: Lymphocytes
NEUTR: Neutrophils
SYN: Synoviocytes.
